# Telomere and Telomerase-Associated Proteins in Endometrial Carcinogenesis and Cancer-Associated Survival

**DOI:** 10.3390/ijms23020626

**Published:** 2022-01-06

**Authors:** Lucy Button, Bryony Rogers, Emily Thomas, Alice Bradfield, Rafah Alnafakh, Josephine Drury, Dharani K. Hapangama

**Affiliations:** 1 Centre for Women’s Health Research, Department of Women’s and Children’s Health, Institute of Life Course and Medical Sciences, University of Liverpool, Liverpool L8 7SS, UK; lucyfbutton@gmail.com (L.B.); B.C.V.Rogers@student.liverpool.ac.uk (B.R.); E.L.Thomas@liverpool.ac.uk (E.T.); R.A.A.Alnafakh@liverpool.ac.uk (R.A.); jadrury@liverpool.ac.uk (J.D.); 2 Liverpool Women’s NHS Foundation Trust, Liverpool L8 7SS, UK; A.J.Bradfield@student.liverpool.ac.uk

**Keywords:** telomere, telomerase, protein, endometrium, endometrial cancer, qPCR, immunohistochemistry

## Abstract

Risk of relapse of endometrial cancer (EC) after surgical treatment is 13% and recurrent disease carries a poor prognosis. Research into prognostic indicators is essential to improve EC management and outcome. “Immortality” of most cancer cells is dependent on telomerase, but the role of associated proteins in the endometrium is poorly understood. The Cancer Genome Atlas data highlighted telomere/telomerase associated genes (TTAGs) with prognostic relevance in the endometrium, and a recent in silico study identified a group of TTAGs and proteins as key regulators within a network of dysregulated genes in EC. We characterise relevant telomere/telomerase associated proteins (TTAPs) NOP10, NHP2, NOP56, TERF1, TERF2 and TERF2IP in the endometrium using quantitative polymerase chain reaction (qPCR) and immunohistochemistry (IHC). qPCR data demonstrated altered expression of multiple TTAPs; specifically, increased *NOP10* (*p* = 0.03) and reduced *NHP2* (*p* = 0.01), *TERF2* (*p* = 0.01) and *TERF2IP* (*p* < 0.003) in EC relative to post-menopausal endometrium. Notably, we report reduced NHP2 in EC compared to post-menopausal endometrium in qPCR and IHC (*p* = 0.0001) data; with survival analysis indicating high immunoscore is favourable in EC (*p* = 0.0006). Our findings indicate a potential prognostic role for TTAPs in EC, particularly NHP2. Further evaluation of the prognostic and functional role of the examined TTAPs is warranted to develop novel treatment strategies.

## 1. Introduction

Endometrial cancer (EC) is the most common gynaecological malignancy [1], responsible for 2500 deaths in the UK in 2018 alone. Despite improvement in 5-year survival over the last 40 years to 75.6% (2013–2017) [2], the risk of recurrence in surgically treated EC is 13% [3]. Unfortunately, the prognosis for recurrent disease is particularly poor; therefore, research into prognostic markers is essential to improve clinical outcomes by informing management decisions [4].

**Figure 1:**
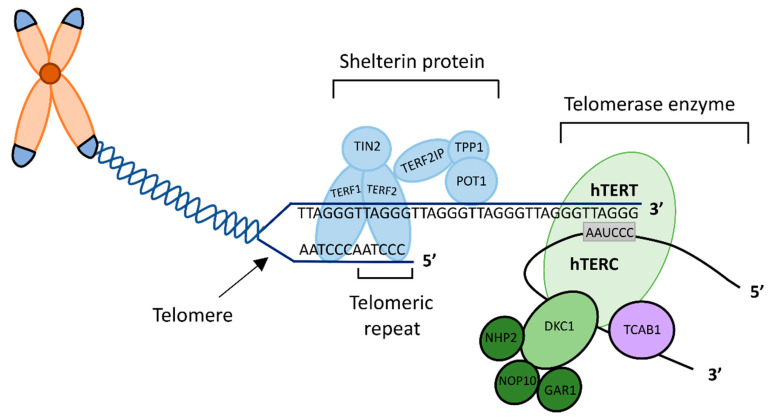
The Structure of the human telomere and telomerase. Telomere is composed of tandem repeats of nucleotide sequence TTAGGG with a single stranded G-rich 3′ overhang. The six-protein complex shelterin or telosome protects telomere ends. TERF1, TERF2 and POT1 directly bind to the telomere interconnected by TERF2IP, TIN2 and TPP1. Telomerase is comprised of three main components hTERT, hTERC and DKC1. hTERC harbours an H/ACA motif which is associated with DKC1 and small nucleolar RNPs NOP10, NHP2 and GAR1.

Telomeres cap the terminal ends of eukaryotic chromosomes and are composed of tandem repeats of the nucleotide sequence TTAGGG [5], which are essential for genomic stability. The telomere DNA sequence has a guanosine rich 3′ overhang which may also act to protect the chromosome from degradation [6,7] (Figure 1). Telomeres are transcribed as long non-coding RNAs called TERRAs (TElomeric Repeat containing RNA), which participate in a variety of cellular regulatory functions [8,9]. With normal cell division, there is loss of telomeric DNA and attrition of the telomere, resulting in an end replication problem [10]. Once the telomere is at a critical length, there is cell cycle arrest through initiating either cellular senescence or apoptosis pathways [11]. Telomeric shortening and dysregulation are implicated with ageing and disease, including cancer [12], thus telomere maintenance (TM) is an essential cellular function.

The specialised ribonucleoprotein complex, telomerase, maintains telomeres through elongation of the single telomeric DNA strand using an RNA template specific to the 3′ sequence to add G-rich repeats [13,14]. Telomerase is a holoenzyme consisting of primary components: human reverse transcriptase (hTERT, the catalytic unit), and the integral human telomerase RNA component (hTERC) [14,15]. hTERC harbours an H/ACA domain essential for 3′ end processing and accumulation of hTERC as well as telomerase activity [16]. This H/ACA motif is responsible for post-transcriptional modifications, and is shared with small nucleolar and small Cajal body RNAs [17]. The motif associates with four core preserved proteins: dyskerin (DKC1), NHP2, NOP10 and GAR1; forming small nucleolar ribonucleoproteins (snoRNP) [17,18,19] (Figure 1). These snoRNPs are responsible for the stability and accumulation of telomerase RNA, which is vital for normal telomerase activity [20]. NOP56 is another snoRNP, which forms a component of the Box C/D snoRNP complex [21]. It is thought to be required for protein synthesis and cellular division as it is involved in pre-rRNA processing and ribosome biogenesis, and is rate limiting for cell proliferation [22,23,24]. NOP56 is indirectly associated with telomerase activity, as it interacts with multiple components of the telomerase complex, including DKC1 and NHP2 [25,26]. Furthermore, hypomorphic mutations within the NOP56 gene may elongate telomeres [27].

The telosome or shelterin, is another protein complex directly associated with the telomere, with a high specificity for the telomeric sequence. The complex consists of six telomere specific proteins; the three subunits Telomeric Repeat factor 1 and 2 (TERF1, TERF2) and Protection of Telomeres 1 (POT1) bind directly to the chromosomal end. Three additional proteins Repressor/Activator Protein 1 (TERF2IP), TERF1-Interacting Nuclear Protein 2 (TIN2) and Tripeptidyl Peptidase 1 (TPP1) interconnect these subunits. Shelterin acts to protect chromosomes from fatal end to end or sister chromatid fusion and deterioration [28] and regulates telomere length through its action on telomerase. TERF2 encourages the formation of telomerase or t-loops, where the terminus of telomeric DNA is ‘tucked’ away. This protects normal linear DNA at the ends of the chromosomes, making them inaccessible, even to telomerase [29]. Consequently, this prevents chromosomal ends from being detected as a double strand break, averting a DNA damage response (DDR) [6], while also suppressing telomerase activity, thus regulating telomere length [29]. TERF1 also regulates telomerase activity through a negative feedback mechanism, stabilising telomere length [30,31].

Telomerase activity is essential for the maintenance of critical telomere length, enabling cell replicative immortality, one of the hallmarks of cancer [32]. Approximately 85% of human cancers achieve indefinite cell proliferation and avoidance of telomeric shortening through increased telomerase activity. In the absence of telomerase, the remainder achieve immortalisation through a different mechanism; the alternative lengthening of telomeres (ALT); considered to primarily involve DNA recombination [33]. Telomerase activity is usually undetectable in normal somatic cells; in contrast to the functionally significant levels observed in approximately 90% of immortalised cells, including cancer cells [32,34]. Nevertheless, the human endometrium is a dynamic somatic organ with a unique ability to undergo monthly regeneration and proliferation [35,36,37]. Previous research has demonstrated high telomerase activity in the premenopausal endometrium, with a transition to low or undetectable levels in the postmenopausal endometrium [36,38,39]. Despite unique endometrial telomerase biology, there is reactivation of telomerase activity from the postmenopausal state to EC [28,29,35,40]. Hence, telomerase must factor in the malignant transformation of the endometrium. Evidently, telomere and telomerase associated proteins (TTAPs) are essential for TM, which is central to cancer cell proliferation. Therefore, these proteins may hold clinical significance in EC.

We hypothesised that TTAPs have a role in the human endometrium and may hold clinical relevance in EC. The specific proteins under examination were selected considering the publically available survival analysis data of The Cancer Genome Atlas (TCGA) EC mRNA sequencing dataset from the Human Protein Atlas, which identified genes with prognostic relevance ( Supplementary Figure S1) [41] and our previously published in silico study [42]. In this ex vivo study, we tested this hypothesis by studying the levels of the genes *NOP10, NHP2, NOP56, TERF1, TERF2* and *TERF2IP* and the proteins they encode, in the healthy human endometrium and in a cohort of EC samples. Subsequently, we assessed the prognostic relevance of the protein immunoscores.

## 2. Results

### 2.1. Patient Population and Demographic Details

A total of 103 patients were included, with 13 pre-menopausal, 27 post-menopausal and 63 with endometrial cancer. Biopsies were obtained for quantitative polymerase chain reaction (qPCR) and/or immunohistochemical analysis. See Table 1 (and Supplementary Table S1) for patient demographics. Within the cohort, previous tamoxifen use was noted in two post-menopausal patients (7%) and two carcinosarcoma samples (13%), in addition to prior use of anastrazole in one (5%) Grade 3 endometrioid cancer patient.

### 2.2. Rationale for Selecting Specific TTAPs

In this study, the specific proteins under examination were selected initially following survival analysis of EC mRNA sequencing dataset of TCGA from the Human Protein Atlas, which identified genes with prognostic relevance (Supplementary Figure S1) [41,43]. TCGA dataset revealed the encoding genes for TTAPs dyskerin, *NOP10*, *NHP2* and wider associated protein *NOP56* to have prognostic relevance in EC (see Supplementary Figure S1) [41,43]. The expression of the *DKC1* gene and protein product dyskerin, have already been thoroughly investigated in the endometrium [44]. The remaining H/ACA snoRNP, GAR1, though seen to be highly expressed, was not prognostic in the endometrium [41,45]; therefore, it was not examined.

Further proteins under examination were selected following our previously published detailed in silico study investigating the role of telomere and telomerase associated genes and proteins in EC. Telomerase associated protein NHP2 and wider associated protein NOP56 were identified as key regulatory genes within a network of genes dysregulated in stage I and IV EC [42], (Supplementary Figure S2 [46]) therefore both were examined.

TERRAs have been seen to be significantly altered in EC [9] and shelterin proteins TERF1/2 are known to interact with TERRA [8]. Thus, we also selected shelterin proteins TERF1/2 for examination. The third shelterin subunit, POT1 was not included as it has already been examined in the endometrium [47]. TERF2IP was included in our study as it binds to TERF2, therefore is structurally relevant [48]. TERF1/2 and TERF2IP proteins which were non-prognostic according to TCGA data, they were also included for study validation. Supplementary Figure S3 demonstrates a protein–protein interaction network diagram created using STRING [25,46]; to explore and identify the predicted functional interaction networks between the proteins of interest.

### 2.3. qPCR Data

**Figure 2:**
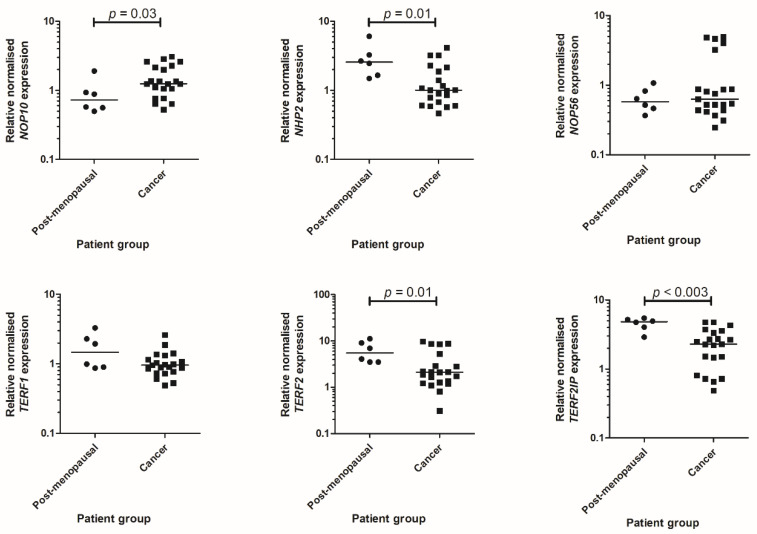
qPCR data for selected telomere and telomerase associated transcripts *NOP10*, *NHP2*, *NOP56*, *TERF1*, *TERF2* and *TERF2IP*. Individual points shown are the mean of technical triplicates, with the median indicated by the line. Post-menopausal, *n* = 6; Cancer, *n* = 21 for all genes examined.

In our independent patient sample set, initially, we determined mRNA expression levels of genes encoding the TTAPs of interest in PM and cancer samples. Rationale for this qPCR analysis was to confirm whether similar differences observed in the TCGA cohort was present in our patient cohort, prior to investigating the protein products of the selected TTAP genes with immunohistochemistry (IHC). We found a statistically significant increase in *NOP10* mRNA expression in cancer tissue relative to healthy post-menopausal endometrium (*p* = 0.03) (Figure 2). *NHP2* (*p* = 0.01), *TERF2* (*p* = 0.01) and *TERF2IP* (*p* < 0.003) mRNA expression was significantly reduced in EC when compared to post-menopausal endometrium. There was no significant alteration in mRNA expression levels for *NOP56* or *TERF1*.

### 2.4. Characterisation of Telomere and Telomerase Associated Protein Expression

**Figure 3:**
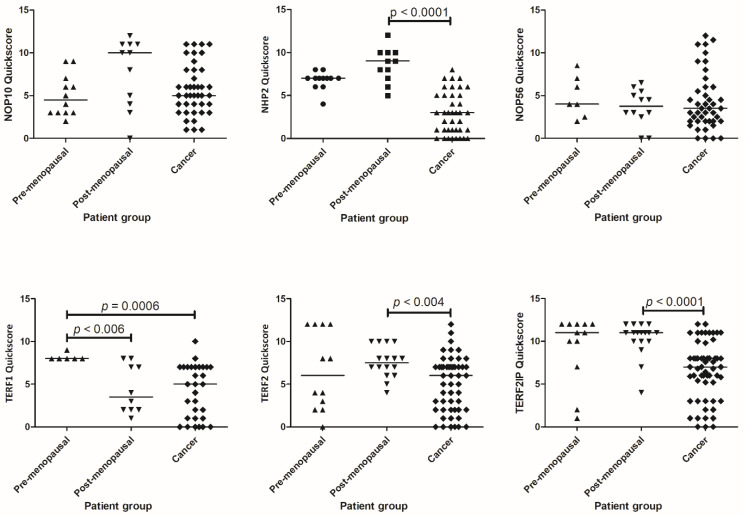
Scatterplots to show Quickscores for NOP10, NHP2, NOP56, TERF1, TERF2 and TERF2IP. Line indicates median. NOP10 pre-menopausal *n* = 12, post-menopausal *n* = 11 (pre-menopausal vs. postmenopausal *p* = 0.059), cancer *n* = 39; NHP2 pre-menopausal *n* = 12, post-menopausal *n* = 11, cancer *n* = 41; NOP56 pre-menopausal *n* = 7, post-menopausal *n* = 12, cancer *n* = 43; TERF1 pre-menopausal *n* = 6, post-menopausal *n* = 10, cancer *n* = 30; TERF2 pre-menopausal *n* = 12, post-menopausal *n* = 16, cancer *n* = 51; TERF2IP pre-menopausal *n* = 12, post-menopausal *n* = 17, cancer *n* = 57.

**Figure 4:**
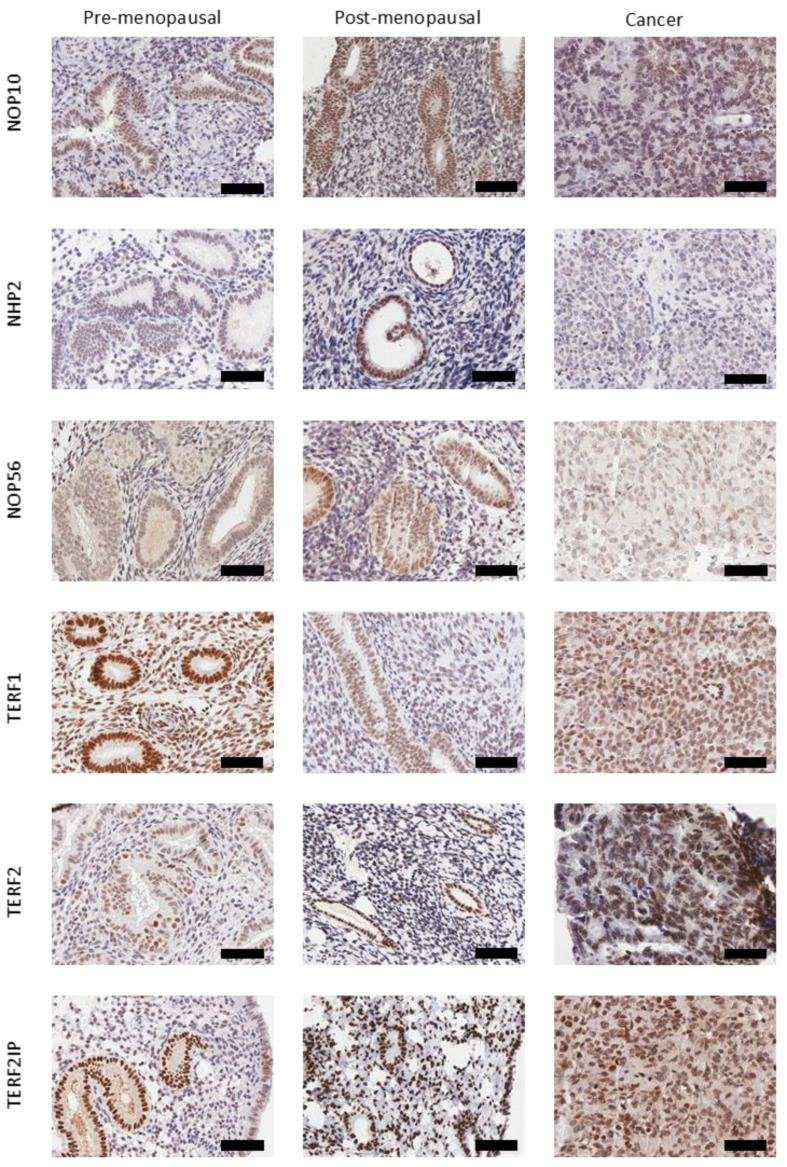
Representative photomicrographs of NOP10, NHP2, NOP56, TERF1, TERF2 and TERF2IP immunohistochemical staining in human endometrial samples from pre-menopausal, post-menopausal and cancer patients. Magnification 40×, scale bar is 60 microns. Positive staining appears brown.

The presence of each protein of interest was visualised at a cellular level using IHC, and immunostaining was semi-quantified using a modified Quickscore in our independent patient sample set. Nuclear staining was assessed in glandular epithelial cells from all endometrial tissue samples. Immunoscores for each protein were compared between healthy pre-menopausal and post-menopausal endometrium and cancer samples (Figure 3); representative micrographs for all studied proteins are illustrated in Figure 4.

We found a statistically significant reduction in NHP2 immunoscores in EC samples relative to healthy post-menopausal endometrium (*p* < 0.0001) (Figure 3). Immunohistochemical staining demonstrated a statistically significant difference in the abundance of TERF1, with reduced Quickscores in post-menopausal (*p* < 0.006) and EC (*p* = 0.0006) tissues relative to healthy pre-menopausal samples. A statistically significant loss of TERF2 and TERF2IP immunostaining was also identified in EC samples when compared to post-menopausal samples (*p* < 0.004 and *p* < 0.0001, respectively).

No significant difference was observed in the abundance of NOP10 or NOP56 between pre-menopausal, post-menopausal and EC samples (Figure 3).

### 2.5. Survival Analysis

**Figure 5:**
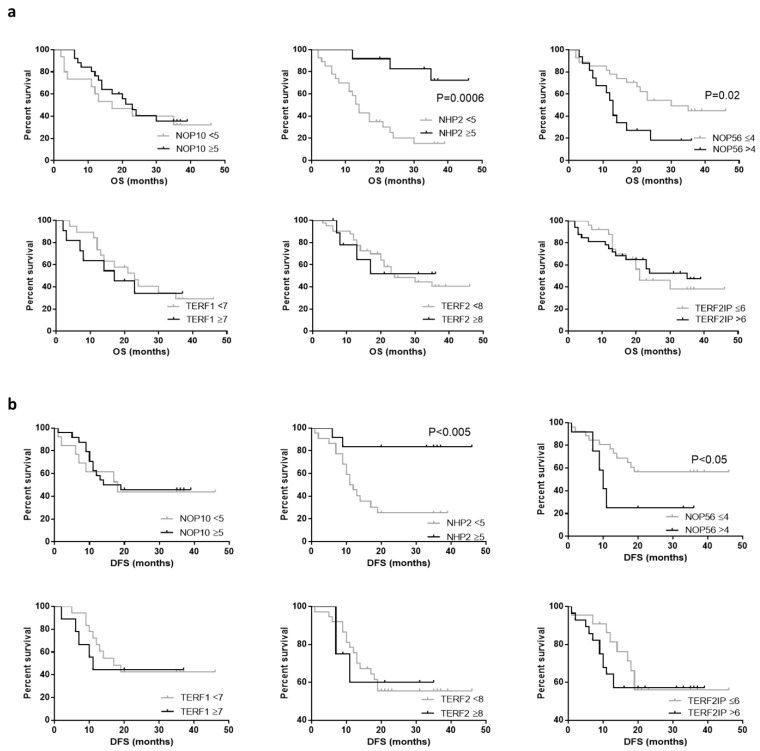
Survival curves obtained from IHC Quickscores of experimental data. Low quickscore data shown in grey, high quickscore data in black. (**a**) Overall survival (OS). Low NOP10 *n* = 15, high NOP10 *n* = 26, *p* = 0.56; low NHP2 *n* = 27 median survival 14 months, high NHP2 *n* = 12, median survival undefined, *p* = 0.0006; low NOP56 *n* = 27, median survival 30 months, high NOP56 *n* = 16 median survival 16 months, *p* = 0.02; low TERF1 *n* = 19, high TERF1 *n* = 11, *p* = 0.78; low TERF2 *n* = 41, high TERF2. *n* = 11, *p* = 0.97; low TERF2IP *n* = 25, high TERF2IP *n* = 33, *p* = 0.69. (**b**) Disease-free survival (DFS). Low NOP10 *n* = 15, high NOP10 *n* = 26, *p* = 0.8; low NHP2 *n* = 27 median DFS 11.5 months, high NHP2 *n* = 12, median DFS undefined, *p* < 0.005; low NOP56 *n* = 27, median DFS undefined, high NOP56 *n* = 16 median DFS 10 months, *p* < 0.05; low TERF1 *n* = 19, high TERF1 *n* = 11, *p* = 0.69; low TERF2 *n* = 41, high TERF2. *n* = 11, *p* = 0.96; low TERF2IP *n* = 25, high TERF2IP *n* = 33, *p* = 0.70.

We performed survival analysis with corresponding survival curves using an optimal cut off for each of the protein IHC Quickscores with clinical outcomes from our local patient cohort as displayed in Figure 5. Survival analysis demonstrated a significant difference between high and low Quickscores of NHP2 (*p* = 0.0006) and high Quickscore of NHP2 was favourable (HR 4.38, 95% CI 1.89–10.16). In our patient cohort, a significant difference was observed in survival when comparing the low and high abundance of NOP56 (*p* = 0.02), where a high abundance of NOP56 was unfavourable (HR 0.35, 95% CI 0.14–0.86). We found no significant difference in survival curves for NOP10, TERF1, TERF2 or TERF2IP.

### 2.6. Correlations

Immunoscores for the proteins of interest for the entire sample cohort were correlated with cell proliferation marker Ki67, and between each protein of interest. Semi-quantitative immunostaining data for the four steroid receptors (ERα, ERβ, PR and AR) in EC samples was correlated with Quickscores for the proteins of interest.

NHP2 (r = −0.45, *p* = 0.001) and TERF2IP (r = −0.42, *p* < 0.0002) protein levels as demonstrated using IHC immunoscores, showed a negative association with Ki67 (see Table 2).

When comparing Quickscores for proteins of interest in healthy and malignant samples (Table 3), we identified statistically significant positive correlations between NHP2 and TERF1 (r = 0.34, *p* = 0.02), NOP56 and TERF2 (r = 0.53, *p* < 0.0002), TERF1 with TERF2 (r = 0.65, *p* < 0.0001) and TERF2IP (*p* = 0.002), TERF2 and TERF2IP (*p* < 0.0001).

When correlating steroid receptor data with immunoscores for the proteins of interest in EC samples, we found a statistically significant positive correlation between PR and NHP2 (r = 0.51, *p* = 0.002) and AR with NHP2 (r = 0.4, *p* = 0.025). We also found a negative association between ERα and NOP56 (r = −0.36, *p* = 0.03) and AR with NOP56 (r = −0.41, *p* = 0.02). There were no significant associations with ERβ (see Table 4).

## 3. Discussion

We identified altered *NOP10*, *NHP2*, *TERF2* and *TERF2IP* mRNA levels and significantly different protein immunostaining levels for NHP2, TERF2 and TERF2IP in EC when compared with healthy post-menopausal endometrium. Both NHP2 and NOP56 protein immunoscores were associated with patient survival, suggesting a prognostic relevance. To our knowledge, this is the first study comprehensively examining and characterising the expression of six TTAPs with purported importance in the healthy and malignant endometrium. We also describe the changes in expression of these six TTAPs in endometrial carcinogenesis, and cancer associated survival.

### 3.1. NOP10

Expression of protein encoding gene *NOP10* has been reported to be decreased in patients with chronic lymphocytic leukaemia (CLL) [49]. Conversely, our qPCR data for *NOP10* demonstrated altered mRNA expression in EC with a statistically significant increase when compared to the post-menopausal endometrium. However, the differential expression of *NOP10* mRNA was not associated with increased levels of endometrial NOP10 protein. We scored nuclear staining and NOP10 is expected to be nucleolar. We assessed nuclear staining because it was difficult to evaluate nucleolar staining when there was also nuclear staining present; nucleolar scoring may be more reflective of mRNA data. Elsharawy et al. examined NOP10 using IHC in breast cancer and reported a significant increase in NOP10 when assessing both nuclear and prominent nucleolar staining. High abundance of NOP10 protein whether in the nucleus or in the nucleoli demonstrated a significant association with unfavourable characteristics including high tumour grade, thus substantiating this hypothesis for non-significance in our study [50].

Post-transcriptional regulation may also be responsible for this discordant relationship, therefore differential *NOP10* mRNA expression may not manifest as variation at the functional level of the protein. Additionally, changes in NOP10 protein levels did not carry any significance for patient outcome, in contrast with TCGA RNA data [43].

### 3.2. NHP2

*NHP2* mRNA expression decreased in EC when compared to post-menopausal endometrium. IHC data revealed a concordant, statistically significant reduction in NHP2 abundance in EC when compared to pre-menopausal and post-menopausal endometrium controls. Previous studies have explored the expression and clinical significance of NHP2 in various cancer types. NHP2 and its encoding gene have been shown to be overexpressed in gastric and colorectal cancers [51,52]. Kim et al., reported increased levels of NHP2 protein and upregulation of its encoding gene in gastric and colorectal cancer tissues [51]. Their contrasting results when compared with our findings may be due to the discrepancies in sample size reducing the generalisability of results in different populations, as well as the biological differences between different cancer types. This possibility needs to be explored further since the human endometrium has been demonstrated to have a unique telomerase biology. High telomerase activity is observed in most epithelial cancers; however, the benign endometrium also demonstrates high telomerase activity [36]. Thus, endometrial carcinogenesis may result in altered function and subsequently altered levels of its associated proteins relative comparison to other malignancies; further functional analysis is thus warranted to explore this. Witkowska et al. identified significant upregulation of *NHP2*, specifically in high stage colonic cancer. Thus, an explanation for their contradicting results may be the selection of samples, with a high clinical stage presenting a confounding variable. Additionally, the use of microarray, a different technique to analyse gene expression, may also affect results.

EC is typically a disease of the post-menopausal endometrium with 90% of cases occurring in women over the age of 50 [53], thus establishing healthy post-menopausal samples as the most appropriate control. The reduction observed in *NHP2* mRNA expression and protein product NHP2 in EC relative to post-menopausal samples is our most notable finding; these results suggest loss of NHP2 is a malignant transformation, indicating NHP2 may have a prospective role as a biomarker.

Further to this, our survival analysis suggests highly abundant NHP2 is favourable for patient outcome in EC. Thus, NHP2 may also have potential as a prognostic indicator, although further research is warranted. Conversely, TCGA data previously demonstrated that high expression of *NHP2* is unfavourable [43]. The disagreement in results may be attributable to contrasting techniques for analysis (TCGA survival analyses use RNA sequencing data), the inclusion of varied cancer types within this study and the use of different ‘normal controls’ in each study. As previously described by Alnafakh et al., TCGA utilises ‘normal’ tissue within 2 cm from the tumour for comparison, in contrast to the external healthy controls within this study. It is vital to consider that endometrioid and serous ECs within the TCGA data set are likely to have originated from a background of endometrial hyperplasia or endometrial intraepithelial neoplasia. Consequently, the control samples obtained from tissue adjacent to the tumour site (considered to be normal endometrium for comparison in TCGA data), may include areas of endometrial hyperplasia or endometrial intraepithelial neoplasia [44]. Additionally, we consider our data to be more generalizable, with the use of histologically confirmed external healthy controls from a well-characterised population.

Furthermore, we propose NHP2 as a promising potential biomarker and possible prognostic indicator, utilising an affordable and accessible technique of immunohistochemical staining. It is important to consider that although a greater abundance of immunologically reactive NHP2 protein is seen within healthy tissues, this protein may not be functional; this can only be determined by functional assays. In this study, we report a statistically significant negative association between NHP2 and cell proliferation marker Ki67, indicating NHP2 loss may occur due to sustained cell proliferation signalling, one of the hallmarks of cancer [32].

The loss of NHP2 observed in EC is interesting when considered in the context of our current understanding of endometrial telomerase biology. Telomerase activity is reported to be at functionally significant levels in the pre-menopausal endometrium [38,39] and EC [28,36]; therefore, a similar expression of TTAPs in EC is expected. Nevertheless, NHP2 demonstrates the contrary, indicating there may be protein encoding gene dysregulation due to disease pathogenesis. Gene expressional analysis is necessary to validate this conclusion.

Estrogen receptor (ER) and progesterone receptor (PR) are the most validated prognostic biomarkers for EC; the loss of both hormone receptors is a predictor of poor prognosis [54,55]. Our data indicate a statistically significant positive association between NHP2 and PR, prompting further investigation into its role in improving the performance of known prognostic indicators. Progesterone inhibits telomerase activity and hTERT expression in the endometrium, and expression of PR has been positively correlated with a good prognosis of EC. ECs regress in response to progesterone, therefore progesterone is used to treat advanced ECs. Telomerase is an indirect downstream target of progesterone [36]. The positive relationship identified between PR and telomerase associated protein NHP2 may imply a greater abundance of NHP2 is associated with a better prognosis, corroborating our survival analysis.

### 3.3. NOP56

*NOP56* has been shown to be upregulated in acute myeloid leukaemia and may also be associated with poorer outcome [56]. It has also been identified as a marker of unfavourable prognosis in liver cancer, renal cancer and melanoma [57,58]. NOP56 is less directly associated with telomerase as it interacts with DKC1 and NOP10, and it is predicted to bind to NHP2 [59,60,61,62]. We found no significant difference in *NOP56* mRNA expression or protein abundance between healthy and malignant endometrium; however, survival analysis indicates high abundance is unfavourable for clinical outcome in EC, in keeping with findings from previous studies in other malignancies [57,58]. NOP56 IHC EC data also showed a negative correlation with steroid receptors ERα and AR, indicating possible significance prognostically, warranting further exploration.

### 3.4. TERF1

Expression of *TERF1* has previously been shown to have significant overexpression or downregulation, dependent on cancer type [63,64,65,66,67]. Interestingly, a study by Bejarano et al. has also validated TERF1 inhibition in glioblastoma as an effective therapeutic strategy [67]. In the endometrium, we report no significant difference in the expression of *TERF1* mRNA or protein levels when comparing post-menopausal endometrium and EC. We identified changes in protein levels when comparing pre-menopausal samples to post-menopausal and EC samples; the significance of this loss of protein in EC is unknown. Since high telomerase activity is a feature of endometrial cancer [28,36], an increased abundance of relevant proteins is anticipated. However, TERF1 is associated with the telomere complex and the protection of telomere length. Despite high telomerase activity, EC cells have been reported to possess shorter telomeres [9] and since TERF1 imposes a negative feedback on telomerase activity [30,31], we can speculate that the observed loss of this protein may be beneficial for maintaining high telomerase activity in EC cells. Survival analyses for TERF1 data were non-significant, and predictable positive correlations were seen with structurally related proteins TERF2 and TERF2IP.

### 3.5. TERF2 and TER2IP

Previous reports have implied abnormally high or low expression of TERF2 may lead to chromosomal instability and induce fatal tumour changes [68]. We observed loss of *TERF2* mRNA and protein levels in EC relative to postmenopausal endometrium, however, TERF2 levels did not carry clinical significance in survival analysis. TERF2 immunoscores positively correlated with TERF2IP, likely due to their structural relationship [28]. Loss of TERF2 may make the telomeres accessible to telomerase, facilitating telomerase activity at the telomere site [29]. TERF2 may hold potential as a biomarker in EC, as reduced protein levels may be attributed to malignant transformation; nevertheless, this needs further exploration. Upregulation of *TERF2* has been previously observed in hepatocellular carcinoma [69] and cervical cancer where it has a positive impact on survival in patients with advanced stage cancer [70].

Similarly, to structurally related protein TERF2, *TERF2IP* mRNA expression and proteins levels are reduced in EC relative to healthy postmenopausal endometrium. TERF2IP EC immunoscores had a negative association with Ki67 thus we anticipated that to indicate functional or prognostic relevance, yet, no significant separation was observed in survival analyses comparing high vs. low protein levels. Contrastingly, *TERF2IP* expression has previously been reported to be significantly higher in breast tumour tissues relative to adjacent non-tumour tissues [71], and highly expressed in colorectal cancer tissues; with high expression correlated with poor prognosis and distant metastasis in colorectal cancer [72]. The alteration in mRNA and protein levels in EC relative to postmenopausal endometrium may implicate TERF2IP as another TTAP with potential as a prognostic indicator, possibly in combination with TERF2, that we were unable to identify in our sample size, therefore further investigation is warranted.

### 3.6. Limitations

Limited sample size due to our modest patient cohort, pose an obstacle for drawing definitive conclusions for the proteins under examination; significant results on such a small scale may not be representative of the population. Though we thoroughly explore the presence of the selected TTAPs in healthy and malignant endometrium, we also acknowledge our methods do not assess the functionality of the proteins under examination. Consequently, we propose future research into the functional and prognostic role for the selected TTAPs. Another limitation is the use of Quickscoring; a semi-quantitative method where subjectivity may influence results. Nevertheless, a subset of samples was double-scored, and scorers met to review any discrepancies and agreed upon final values.

## 4. Materials and Methods

A selection of TTAPs from both the telomerase and shelterin complexes and associated proteins were chosen to examine their expression using real time quantitative reverse transcriptase PCR (qPCR) and immunohistochemistry (IHC) in endometrial biopsies obtained from women with EC and post-menopausal women with no endometrial abnormalities.

Ethical approval was granted by Liverpool and Cambridge Adult Research Ethics Committee (LREC 09/H1005/55, 11/H1005/4 and CREC 10/H0308/75) and informed written consent was obtained from all participants.

### 4.1. Patient Population and Sample Collection

Endometrial biopsies were obtained from women undergoing surgery in Liverpool Women’s Hospital between 2009 and 2014 [73]. Samples were divided and either (i) fixed (≥24 h in 10% (*v/v*) neutral buffered formalin) and paraffin embedded for immunohistochemical staining, or (ii) placed in RNALater.

Adhering to the International Federation of Gynaecology and Obstetrics guidance [20], gynaecological pathologists allocated histological descriptors for EC type and grade. Normal endometrial samples were assigned phases according to histological features and last menstrual date as described by Kamal et al. [21].

Patient clinico-pathological and demographic details were obtained by review of hospital records and clinical databases. Patients included in the study did not receive any neoadjuvant hormonal treatment, chemotherapy or radiotherapy.

### 4.2. qPCR

qPCR was conducted as previously described [9,44,74]. Briefly, total RNA from tissue samples and cultured cell lines were extracted using PureLink RNA mini kit (Invitrogen by ThermoFisher Scientific, Loughborough, UK) according to the manufacturer’s instructions. RNA concentration and purity was assessed spectrophotometrically.

DNA was removed using RQ1 RNase-Free DNase (Promega, Southampton, UK) followed by reverse transcription of 1 μg total RNA using iScript cDNA synthesis kit (Bio-Rad, Hertfordshire, UK), and stored at −80 °C. A no-reverse transcriptase (NRT) control was prepared with each reaction.

Gene expression was quantified by qPCR in triplicate using iTaq universal SYBR green supermix and the CFX Connect Real-Time System (Bio-Rad, Hertfordshire, UK) for the three housekeeping genes, *PPIA*, *IPO8* and *MRPL19* (Sigma Custom Products (Merck), Haverhill, Suffolk, UK) and for the genes of interest; *NOP10*, *NHP2*, *NOP56*, *TERF1*, *TERF2* and *TERF2IP* (Bio-Rad, Hertfordshire, UK). The sequences and amplicon length of each gene used are displayed in Table 5.

Amplification was for 40 cycles of denaturation at 95 °C (5 s) and annealing/extension at 60 °C (30 s) after initialisation at 95 °C (2 min). Melt curves were obtained at the end of each run and amplification products were verified using agarose gel electrophoresis to confirm the amplicon length. Standard curves were generated for all genes using the HEK293 cell line.

### 4.3. Immunohistochemistry

Immunohistochemistry was performed as previously described [75,76,77]. Before immunostaining, 3 µm, formalin-fixed, paraffin embedded tissue sections were deparaffinised in xylene and rehydrated through descending concentrations of ethanol before antigen retrieval in a pressure cooker for 2 min at pH 6.0 in 10 mmol/L citrate buffer.

Endogenous peroxidase activity was quenched in 0.3% H_2_O_2_. Sections were incubated with primary antibody, under the conditions detailed in Table 6. Detection was with ImmPRESS HRP polymer-based system and visualisation was achieved using ImmPACT DAB (Vector Laboratories, Peterborough, UK). Samples were counterstained with Gill 2 Haematoxylin (Thermo Shandon, Runcorn, UK) before brief immersion in acid alcohol and dehydration through ascending concentrations of ethanol, clearing in xylene and mounting in synthetic resin (Consul Mount, Thermo Fisher, Runcorn, UK).

As a negative control, non-immune mouse or rabbit IgG (Vector Laboratories, Peterborough, UK) at 3 μg/mL replaced the primary antibody.

Nuclear staining of endometrial glands was analysed semi-quantitatively using a modified Quickscore estimated under light microscopy at a magnification of 20×. As NOP56 is a nucleolar protein, only the nucleolar staining was scored and this was performed at a magnification of 60×. Staining intensity (0 = no staining, 1 = weak, 2 = moderate, 3 = strong) was multiplied by percentage of positive cells (0–25% = 1, 25–50% = 2, 51–75% = 3, >75% = 4) to obtain a final score out of 12. A subset of samples was double-scored independently by a gynaecological pathologist; allocated scores were compared then scorers met to agree on discrepancies.

Available data for the four steroid receptors (ERα, ERβ, PR and AR) expression and cell proliferation marker Ki67 was correlated with study data. The immunostaining for steroid receptors was also assessed semi-quantitatively using a four-tiered Liverpool Endometrial Steroid Quickscore and the Ki67 proliferative index was evaluated as the percentage of immunopositive cells, of any intensity [78,79].

### 4.4. Statistical Analysis

Relative transcript expression was calculated by the ΔΔCT method, relative to the three reference genes, *PPIA, IPO8* and *MRPL19* and normalised to the Ishikawa cell line control, using Bio-Rad CFX manager (Bio-Rad, Hertfordshire, UK).

GraphPad Prism 5 (GraphPad Software; San Diego, CA, USA) was used for all statistical analysis. The non-parametric one-way ANOVA Kruskal–Wallis was used to examine multiple groups. The non-parametric Mann–Whitney U-test was also used to analyse differences between two groups. ECs were categorized according to protein expression scores (high or low abundance) to compare the expression in relation to survival. Several quick score cut-off points were tested (starting with the median) to identify the expression cut-off that distinguished the group with the worst outcomes [79]. Out of the tested cut-offs, the one showing the best categorization was used and is displayed on the graphs in Figure 5. The Mantel-Cox test was used for survival curve comparisons. Spearman’s rank correlation co-efficient was calculated to measure associations between proteins of interest, Ki67 and steroid receptors. For all comparisons, a *p*-value of <0.05 was considered significant.

## 5. Conclusions

In summary, we present a comprehensive description of six different TTAPs in the healthy human endometrium, and in EC samples. In this study we report upregulation of *NOP10* and downregulation of *NHP2*, *TERF2* and *TERF2IP* in EC relative to healthy post-menopausal endometrium. Further to this, IHC analysis highlighted NHP2, TERF2 and TERF2IP immunoscores were also reduced in EC. Our results indicate TTAPs NHP2, TERF2 and TERF2IP have potential as a future biomarker in endometrial carcinogenesis, that can be detected utilising affordable and widely used laboratory techniques. Survival analysis implies NHP2 protein levels may also carry prognostic significance in cancer associated survival, with improved survival in EC with high abundance of NHP2. Our data will inform researchers areas in telomerase and telomere biology to focus future research in EC. Further evaluation of the prognostic and functional role of the examined proteins is warranted to develop novel treatment strategies in EC.

## Figures and Tables

**Table 1:** Patient cohort demographic details.

Patient Group	Number of Patients	Age Median (Range)	BMI Median (Range)	Parity Median (Range)	Smokers Number (%)	HRT Number (%)	Death Number (%)	Recurrence Number (%)
Pre-Menopausal	13	41 (32–57)	26.7 (18.9–40.5)	2 (1–6)	6 (46%)	n/a	n/a	n/a
Post-Menopausal	27	62 (51–85)	24.9 (17.9–39.6)	3 (0–5)	6 (22%)	1 (4%)	n/a	n/a
Cancer	63	68 (37–78)	28.75 (20.2–54.4)	2 (0–7)	3 (5%)	7 (11%)	30 (48%)	30 (48%)

**Table 2:** Ki67 correlations with telomere and telomerase associated protein Quickscores for all samples.

Ki67 Correlations	NOP10	NHP2	NOP56	TERF1	TERF2	TERF2IP
Number	51	48	48	36	70	73
Spearman r	−0.09	**−0.45**	0.04	−0.33	−0.23	**−** **0.42**
95% confidence interval	−0.36 to 0.20	**−0.66 to −0.19**	−0.25 to 0.33	−0.60 to 0.01	−0.45 to 0.01	**−** **0.60 to** **−** **0.21**
*p* value (two-tailed)	0.54	**0.001**	0.77	0.05	0.06	**<0.0002**

Statistically significant results are indicated in bold text.

**Table 3:** Correlations between telomere and telomerase associated protein Quickscores for all samples.

	NOP10	NHP2	NOP56	TERF1	TERF2	TERF2IP
NOP10	r	0.16	0.24	−0.14	0.15	−0.24
	*p*	0.22	0.07	0.35	0.31	0.08
	*n*	59	59	46	51	54
NHP2	0.16	r	−0.12	**0.34**	0.24	0.27
	0.22	*p*	0.39	**0.02**	0.11	0.05
	59	*n*	56	**46**	48	53
NOP56	0.24	−0.12	r	0.13	**0.53**	0.24
	0.07	0.39	*p*	0.41	**<0.0002**	0.09
	59	56	*n*	43	**46**	52
TERF1	−0.14	**0.34**	0.13	r	**0.65**	**0.48**
	0.35	**0.02**	0.41	*p*	**<0.0001**	**0.002**
	46	**46**	43	*n*	**36**	**38**
TERF2	0.15	0.27	**0.53**	**0.65**	r	**0.46**
	0.31	0.05	**<0.0002**	**<0.0001**	*p*	**<0.0001**
	51	48	**46**	**36**	*n*	**76**
TERF2IP	−0.24	0.27	0.24	**0.48**	**0.46**	r
	0.08	0.05	0.09	**0.002**	**<0.0001**	*p*
	54	53	52	**38**	**76**	*n*

R = Spearman rank correlation, *p* = 2-tailed *p* value, *n* = number of pairs analysed. Statistically significant correlations highlighted in bold text.

**Table 4:** Steroid hormone receptor correlations with telomere and telomerase-associated protein quickscores for endometrial cancer samples.

	PR	ERα	ERβ	AR
NOP10				
*n*	36	34	34	34
Spearman r	−0.03	0.25	0.06	−0.08
95% confidence interval	−0.36 to 0.32	−0.10 to 0.55	−0.29 to 0.40	−0.41 to 0.28
*p* value (two-tailed)	0.88	0.15	0.72	0.66
NHP2				
*n*	33	31	31	31
Spearman r	0.51	0.09	0.17	0.40
95% confidence interval	0.20 to 0.73	−0.28 to 0.44	−0.20 to 0.51	0.04 to 0.67
*p* value (two-tailed)	**0.002**	0.63	0.35	**0.03**
NOP56				
*n*	37	35	35	35
Spearman r	−0.30	−0.36	−0.12	−0.41
95% confidence interval	−0.57 to 0.04	−0.62 to −0.02	−0.44 to 0.23	−0.66 to −0.08
*p* value (two-tailed)	0.07	**0.03**	0.49	**0.02**
TERF1				
*n*	27	25	25	25
Spearman r	−0.03	−0.02	−0.39	−0.10
95% confidence interval	−0.41 to 0.37	−0.42 to 0.39	−0.69 to 0.01	−0.49 to 0.32
*p* value (two-tailed)	0.90	0.91	0.05	0.62
TERF2				
*n*	44	42	42	42
Spearman r	−0.14	0.01	−0.05	−0.07
95% confidence interval	−0.43 to 0.17	−0.30 to 0.32	−0.36 to 0.26	−0.37 to 0.25
*p* value (two-tailed)	0.37	0.92	0.73	0.68
TERF2IP				
*n*	49	47	47	47
Spearman r	−0.03	0.07	−0.18	0.21
95% confidence interval	−0.32 to 0.26	−0.23 to 0.36	−0.45 to 0.12	−0.09 to 0.47
*p* value (two-tailed)	0.84	0.65	0.23	0.16

*n* = number of pairs analysed for each correlation. Statistically significant results are indicated in bold text.

**Table 5:** Gene Sequences and amplicon length of all genes examined, *IPO8, PPIA, MRPL19, NOP10, NHP2, NOP56, TERF1, TERF2* and *TERF2IP*.

Gene Name	Sequences (5′–3′)/Unique Assay ID	Amplicon Length
*IPO8*	Forward: AGGATCAGAGGACAGCACTGCA Reverse: AGGTGAAGCCTCCCTGTTGTTC	102
*PPIA*	Forward: AGACAAGGTCCCAAAGAC Reverse: ACCACCTGACACATAAA	118
*MRPL19*	Forward: CAGGAAGAGGACTTGGAGCTAC Reverse: GCTATCATCCAGCCGTTTCTCTA	137
*NHP2*	qHsaCED0037860	164
*NOP10*	qHsaCID0012562	83
*NOP56*	qHsaCEP0025699	89
*TERF1*	qHsaCID0022415	100
*TERF2*	qHsaCID0015690	123
*TERF2IP*	qHsaCID007952	123

**Table 6:** Antibody conditions.

Antibody	Clone	Type and Host Species	Supplier	Dilution	Incubation
NOP10	EPR8856	Rabbit monoclonal	Abcam (Cambridge, UK)	1/2000	O/N 4 °C
NHP2	H-9	Mouse monoclonal	Santa Cruz Biotechnology(Insight Bio, Wembley, UK)	1/8000	O/N 4 °C
				1/2000	30 min RT
NOP56	CL2603	Mouse monoclonal	Abcam (Cambridge, UK)	1/1000	O/N 4 °C
TERF1	TRF-78	Mouse monoclonal	Santa Cruz Biotechnology(Insight Bio, Wembley, UK)	1/50	O/N 4 °C
TERF2	B5	Mouse monoclonal	Santa Cruz Biotechnology(Insight Bio, Wembley, UK)	1/200	O/N 4 °C
TERF2IP	4C8/1	Mouse monoclonal	Santa Cruz Biotechnology(Insight Bio, Wembley, UK)	1/100	O/N 4 °C
Ki67	MM1	Mouse monoclonal	Leica (Milton Keynes, UK)	1/200	O/N 4 °C
ERα	6F11	Mouse monoclonal	Leica (Milton Keynes, UK)	1/50	2 h RT
ERβ	PPG5/10	Mouse monoclonal	Abcam (Cambridge, UK)	1/50	O/N 4 °C
PR	Pgr636	Mouse monoclonal	DAKO (Ely, UK)	1/1000	1 h RT
AR	AR441	Mouse monoclonal	DAKO (Ely, UK)	1/75	O/N 4 °C

## Data Availability

Data is contained within the article or Supplementary Material.

## Supplementary Materials

Supplementary materials can be found at https://www.mdpi.com/article/10.3390/ijms23020626/s1.

## Abbreviations

TTAPs	Telomere and telomerase associated proteins
TCGA	The Cancer Genome Atlas
EC	Endometrial cancer
qPCR	Quantitative polymerase chain reaction
IHC	Immunohistochemistry
