# Supplementary material for: Telomere and Telomerase-Associated Proteins in Endometrial Carcinogenesis and Cancer-Associated Survival
Source: Int J Mol Sci. 2022 Jan 6;23(2):626. doi: 10.3390/ijms23020626 (PMC8775816; doi:10.3390/ijms23020626)
Supplement: Supplementary file 1 [file ijms-23-00626-s001.zip › ijms-1474691-supplementary.pdf]

Supplementary Materials

Table S1. Patient cohort detailed demographic details.

Patient Group	Number of patients	Age median (range)	BMI median (range)	Parity median (range)	Smokers number (%)	HRT number (%)	Death number (%)	Recurrence number (%)
Proliferative Phase	7	43 (32-57)	35.7 (22-40.5)	2 (1-3)	2 (29%)	n/a	n/a	n/a
Secretory Phase	6	41 (37-45)	22.05 (18.9-31.6)	4 (2-6)	4 (67%)	n/a	n/a	n/a
Post-Menopausal	27	62 (51-85)	24.9 (17.9-39.6)	3 (0-5)	6 (22%)	1 (4%)	n/a	n/a
Grade 1 Endometrioid	15	65 (46-88)	29 (21.6-46.1)	2 (0-5)	1 (7%)	0	4 (27%)	3 (20%)
Grade 2 Endometrioid	13	67 (37-86)	27.6 (21-54.4)	0 (0-5)	1 (8%)	2 (15%)	4 (31%)	4 (31%)
Grade 3 Endometrioid	8	69.5 (60-80)	28.6 (23.9-42.7)	2 (0-6)	0	0	4 (50%)	3 (38%)
Endometrioid Carcinoma*	36	67 (37-88)	28.9 (21-54.4)	2 (0-6)	2 (6%)	2 (6%)	12 (33%)	11 (31%)
Clear Cell Carcinoma	4	67 (60-82)	30.25 (26.6-39)	3 (2-4)	0	0	3 (75%)	3 (75%)
Carcinosarcoma	16	71.5 (57-87)	24.2 (20.2-51.4)	2 (0-4)	1 (6%)	4 (25%)	10 (63%)	10 (63%)
Serous	6	75.5 (64-79)	30.4 (27.5-34.8)	3 (2-7)	0	1 (17%)	4 (67%)	5 (83%)
Mixed Clear Cell & Endometrioid	1	82	28	3	0	0	1 (100%)	1 (100%)

Combined data for all grades of endometrioid carcinoma

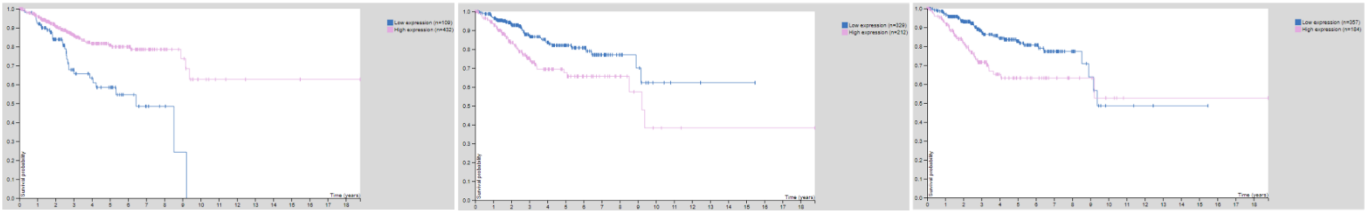

NOP10

High expression favourable, P=0.000015

NHP2

High expression unfavourable, P=0.0005

NOP56

High expression unfavourable, P=0.00012

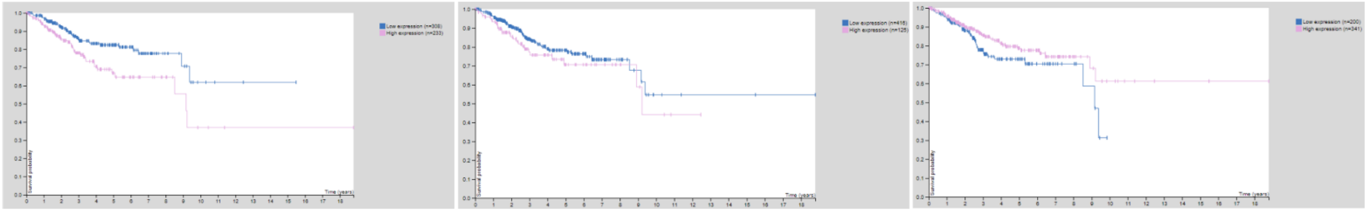

TERF1

Not prognostic, P=0.002

TERF2

Not prognostic, P=0.16

TERF2IP

Not prognostic, P=0.078

Figure S1. Kaplan-Meier survival curves using The Cancer Genome Atlas mRNA sequencing dataset for the proteins of interest, publicly available from the Human Protein Atlas [1] using optimal cut-off. mRNA sequencing data demonstrates *NOP10 [2]*, *NHP2* [3] and *NOP56* [4] are significant prognostically in EC. High expression of *NHP2* and *NOP56* is unfavourable (p=0.0005 and p=0.00012 respectively), while high expression of *NOP10* is favourable (p=0.000015). *TERF1 [5]*, *TERF2 [6]* and *TERF2IP [7]* are not prognostic.

Image credit: The Human Protein Atlas.

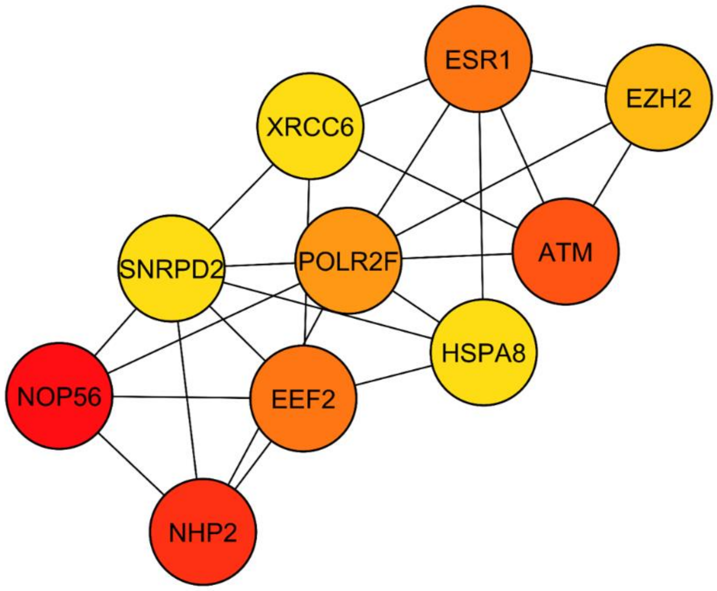

Figure S2. Previous *in silico* study findings [8]. The top 10 hub genes of a protein interaction network constructed from differentially expressed genes in stage I and IV EC, ranked according to degree. Degree represents the number of other genes within the network that they each interact with. Amongst the hub genes, NOP56 and NHP2 had the highest degrees. The colour of the node signifies the degree; red and yellow represent higher and lower degrees respectively. The network was constructed with Cytoscape and hub genes were identified using the Cytohubba plug in.

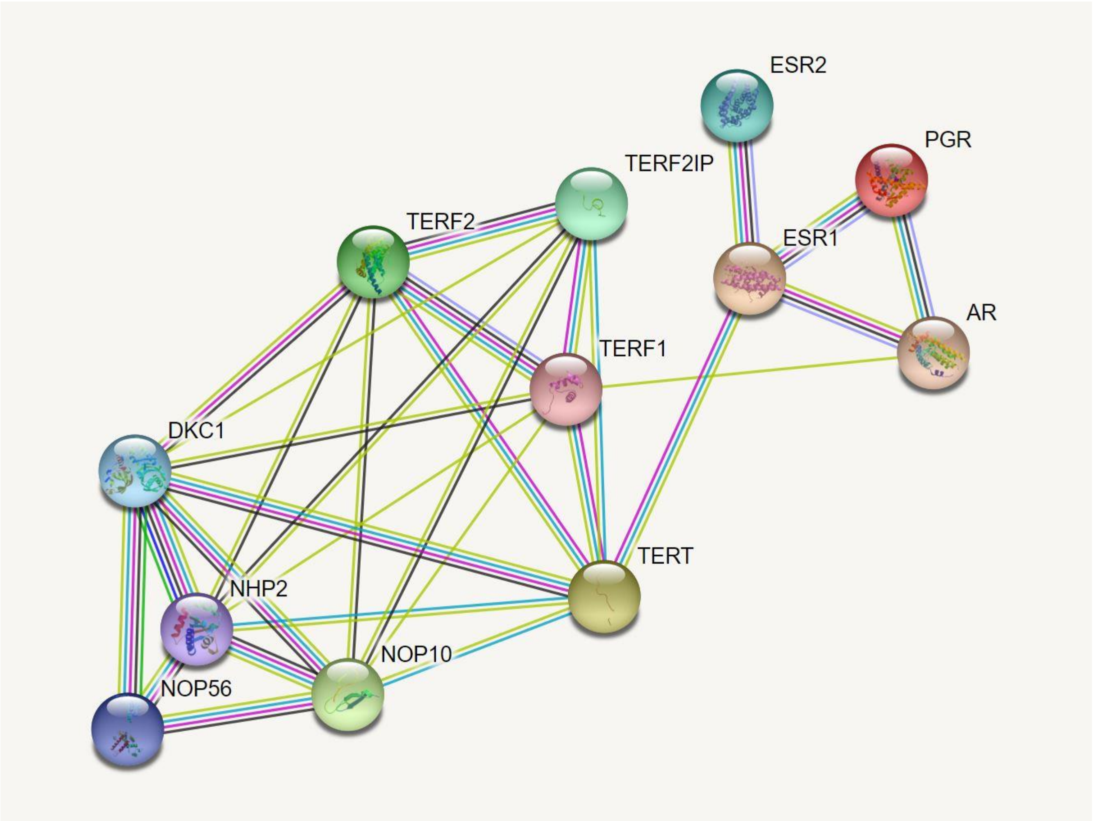

Figure S3. STRING [9,10] network for proteins of interest and related proteins including steroid receptors. ESR1 (ERα), ESR2 (ERβ). Network nodes present proteins, edges represent protein-protein associations.

Node colour: coloured nodes - query proteins and first shell of interactions; white nodes - second shell of interactors.

Node content: filled nodes - some 3D structure is known or predicted.

Colour of edges: turquoise - from curated databases (known interactions); fuchsia - experimentally determined (known interactions); green - gene neighbourhood (predicted interactions); red - gene fusions (predicted interactions); blue - gene co-occurrence (predicted interactions); yellow – textmining; black - co-expression; lilac - protein homology.

References:

1. Uhlen, M.; Zhang, C.; Lee, S.; Sjöstedt, E.; Fagerberg, L.; Bidkhori, G.; Benfeitas, R.; Arif, M.; Liu, Z.; Edfors, F.; et al. A pathology atlas of the human cancer transcriptome. *Science (New York, N.Y.)* **2017**, *357*, doi:10.1126/science.aan2507.
2. The Human Protein Atlas. NOP10 - ENDOMETRIAL CANCER - Interactive survival scatter plot & Survival analysis. Available online: https://www.proteinatlas.org/ENSG00000182117-NOP10/pathology/endometrial+cancer (accessed on 24th December 2021).
3. The Human Protein Atlas. NHP2 - ENDOMETRIAL CANCER - Interactive survival scatter plot & Survival analysis. Available online: https://www.proteinatlas.org/ENSG00000145912-NHP2/pathology/endometrial+cancer (accessed on 24th December 2021).
4. The Human Protein Atlas. NOP56 - ENDOMETRIAL CANCER - Interactive survival scatter plot & Survival analysis. Available online: https://www.proteinatlas.org/ENSG00000101361-NOP56/pathology/endometrial+cancer (accessed on 24th December 2021).
5. The Human Protein Atlas. TERF1 - ENDOMETRIAL CANCER - Interactive survival scatter plot & Survival analysis. Available online: https://www.proteinatlas.org/ENSG00000147601-TERF1/pathology/endometrial+cancer (accessed on 24th December 2021).
6. The Human Protein Atlas. TERF2 - ENDOMETRIAL CANCER - Interactive survival scatter plot & Survival analysis. Available online: https://www.proteinatlas.org/ENSG00000132604-TERF2/pathology/endometrial+cancer#ihc (accessed on 24th December 2021).
7. The Human Protein Atlas. TERF2IP - ENDOMETRIAL CANCER - Interactive survival scatter plot & Survival analysis. Available online: https://www.proteinatlas.org/ENSG00000166848-TERF2IP/pathology/endometrial+cancer (accessed on 24th December 2021).
8. Bradfield, A.; Button, L.; Drury, J.; Green, D.C.; Hill, C.J.; Hapangama, D.K. Investigating the Role of Telomere and Telomerase Associated Genes and Proteins in Endometrial Cancer. *Methods and protocols* **2020**, *3*, doi:10.3390/mps3030063.
9. STRING: functional protein association networks. Available online: https://string-db.org/ (accessed on 14th June 2020).
10. Szklarczyk, D.; Gable, A.L.; Nastou, K.C.; Lyon, D.; Kirsch, R.; Pyysalo, S.; Doncheva, N.T.; Legeay, M.; Fang, T.; Bork, P.; et al. The STRING database in 2021: customizable protein-protein networks, and functional characterization of user-uploaded gene/measurement sets. *Nucleic acids research* **2021**, *49*, D605-d612, doi:10.1093/nar/gkaa1074.
